# Towards a ‘theory of change’ for ocean plastics: a socio-oceanography approach to the global challenge of plastic pollution

**DOI:** 10.1186/s43591-025-00127-8

**Published:** 2025-05-13

**Authors:** Alice A. Horton, Lesley Henderson, Cressida Bowyer, Winnie Courtene-Jones, Samantha L. Garrard, Nieke Monika Kulsum, Deirdre McKay, Imali Manikarachchige, Sreejith Sreekumar, Thomas Stanton

**Affiliations:** 1 National Oceanography Centre, European Way, Southampton, SO14 3ZH UK; 2 Department of Humanities, University of Strathclyde, 16 Richmond St, Glasgow, G1 1XQ UK; 3 Revolution Plastics Institute, University of Portsmouth, Portsmouth, PO1 3DE UK; 4 International Marine Litter Research Unit, School of Biological and Marine Science, University of Plymouth, Drake Circus, Plymouth, PL4 8 AA UK; 5 School of Ocean Science, Bangor University, Menai Bridge, LL59 5 AB UK; 6 Plymouth Marine Laboratory, Prospect Pl, Plymouth, PL1 3DH UK; 7 Department of Communication Sciences, Faculty of Social and Political Sciences, Universitas Nasional Jawa, Barat, 16424 Indonesia; 8 School of Geography, Geology and the Environment, William Smith Building, Keele University, Staffordshire, ST5 5BG UK; 9 Faculty of Engineering and Physical Sciences, University of Southampton, Burgess Road, Southampton, SO16 7QF UK; 10 ICAR-Central Institute of Fisheries Technology, CIFT Junction, Willingdon Island, Matsyapuri P.O, Cochin, 682 029 Kerala India; 11 Geography and Environment, Loughborough University, Loughborough, LE11 3 TU UK

**Keywords:** Plastic Pollution, Social Justice, Environmental Science, Social Science, Citizen Science

## Abstract

Socio-oceanography is an emerging field which mobilises insights from natural and social sciences to explore the inter-connectedness of societal relationships with the ocean and to adopt a holistic approach to solving key oceanographic and societal challenges. It is within this specific context that we explore and reflect upon diverse communities in relation to engaging with plastic pollution in the ocean, one of the foremost socio-environmental challenges of our time. We establish definitions of ‘community’, arguing that communities are not ‘out there’ waiting to be engaged with but are dynamic and (re)constituted in four key contexts - geographical, practical, virtual, and circumstantial. We outline some ‘rules of engagement’ and draw upon several international case studies in the context of plastic pollution to evidence and emphasise the value of working with members of diverse communities to better address socio-oceanographic challenges. In the context of plastic pollution, communities have a vital role to play in terms of co-creating knowledge, lived experience, diverse expertise, and agency to bring about social change. Given the ubiquity of plastics in our day-to-day lives, and subsequently as an environmental pollutant, no community is unaffected by this issue. Relating to socio-oceanography, we argue that structural power imbalances in terms of how diverse communities and natural scientists are traditionally positioned within academic research mean that ‘formal’ scientific knowledge is frequently privileged, and members of communities risk being positioned as ’empty vessels’. Moving away from this ‘deficit’ model where knowledge is simply *transferred* or alternatively *extracted* from communities allows us to progress towards an inclusive ‘socio-oceanography in society’ approach, where members of communities are valued as vital in prioritising and addressing socio-oceanography issues which affect everyday life. Accessibility, openness, ethics and fairness in data are also essential in ensuring that research outcomes can be applied widely outside the academic community.

**Graphical Abstract:**

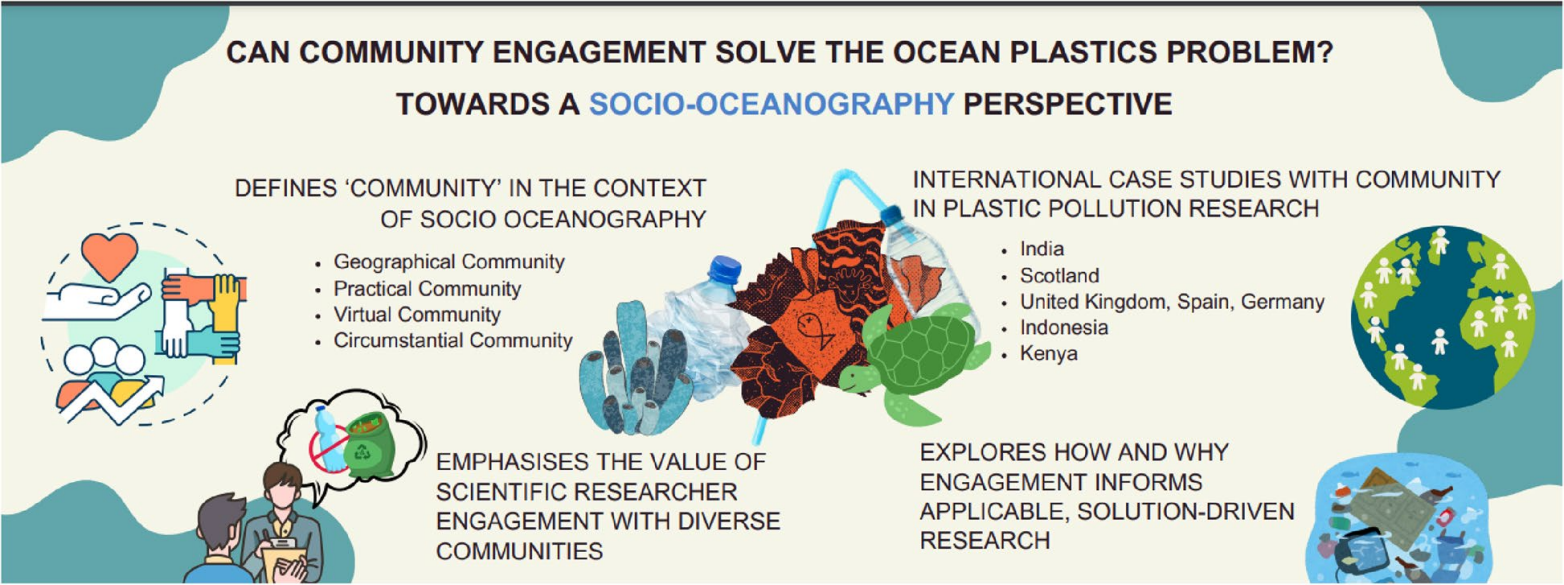

## Introduction

Plastic consumption now outpaces population growth in many parts of the globe [11, 15, 34], with petrochemical corporations seeking to expand their income stream and flood novel markets with plastic [2, 14]. Across the world, a wide range of applications for plastics have been normalised as ‘development’, with their convenience signalling progress, comfort and modernity [2, 12, 24]. Plastics have revolutionised the way we live today, transforming healthcare, transport and communications technology. Without plastics, many of the most important advances of the last decades would not have been possible.

However, many plastics are not designed for long-term transformative use, and unmanageable levels of plastic waste continue to build in the environment. A large proportion of this plastic waste enters the ocean: in 2010 between 4.8 - 12.7 MT was thought to enter the ocean [15], with this estimated to increase to ~29 MT by 2040 [20]. Global mismanaged plastic waste produced within 50 km of the coast is anticipated to double from 2010 to 2025, with 15–40% of that plastic expected to end up as marine debris [15]. The global per capita consumption of plastic is growing, and to date plastic mitigation and regulation measures have been unsuccessful in stemming plastic leakage into the marine environment. Plastics are thus recognised as one of the world’s most intractable socio-environmental challenges [8]. Representatives from 175 countries will meet in Geneva, Switzerland (5–14 August 2025) for the second part of the fifth session (INC-5.2) to negotiate a global treaty to end plastic pollution that comprehensively addresses the full life cycle of plastic, including its production, design, and disposal. While the opportunity to potentially reframe our societal relationship with plastic is the source of significant optimism, the negotiating process has not been without controversy – notably the actual or perceived marginalisation of indigenous communities and scientists in the process to date [40]. Furthermore, negotiations thus far have proven challenging due to the conflicting interests posed by the need to reduce the input and impacts of plastic waste versus the profits of petrochemical companies and the countries that produce these resources [8]. At the time of writing, tangible outcomes of this ongoing process are yet to be realised.

As awareness has risen, public opinion and pressure have been fundamental in shaping plastic pollution discourse and underscoring the necessity for policy interventions. However, a disconnect between research, public perception and action means that in most cases, effective solutions are yet to be implemented. In this paper our goal is to reflect upon how scientific and wider academic engagement with diverse communities may address plastic pollution and its impacts. This paper is informed by an interactive workshop session within the Socio-Oceanography workshop held at the National Oceanography Centre in March 2024 with the deliberately provocative title ‘Can community engagement solve the ocean plastics problem? We recognise the necessity of thinking wider than science into integrating academic research more broadly and invited a mix of environmental and social scientists as well as humanities scholars to participate in this workshop - thus a scientific perspective forms the basis of these discussions. In this paper we also reflect upon a series of international case studies which support our discussion by presenting original empirical data and representing a range of approaches to community.

Communities of various types and scales are already being recognised as key actors in research, advocacy, and policy design for the regulation of ocean plastics. However, amidst a growing recognition of the potential of community engagement in interdisciplinary research, there remain some fundamental questions. Our ‘theory of change’ for ocean plastics posits that the scientific assessment of the problem is vital, yet insufficient in itself to change policies which regulate plastic waste, to reduce and limit the varieties of plastics in the waste stream. Our argument thus begins from the point that plastic pollution is not solely an environmental issue but a social one, and therefore solutions are not possible without an integrated approach. This is supported by the emerging interdisciplinary approach of socio-oceanography which draws together researchers from across the environmental and social sciences to tackle ocean-based lines of inquiry which have yet to be addressed cooperatively [31]. Positive changes to protect the health of our oceans will not simply arise from disseminating the findings of scientific research. Instead, pro-oceans change is predicated on scientists collaborating with each other, researchers from other disciplines, wider communities and lay publics, to integrate diverse spheres of knowledge. Socio-oceanography can thus play a significant role in meeting the considerable range of societal and environmental challenges.

Although the environmental element of socio-oceanography is rooted in marine science, this interdisciplinary approach is applicable across all areas of environmental science (more broadly, socio-environmental research [18, 32]. Social scientists and environmental scientists currently frequently work in silos, adopting different approaches, perspectives, methodological tools and even language. In a time of climatic alterations and natural resources depletion, there is a key requirement for scientific approaches to address ‘real-world’ problems through cooperation across multiple disciplines. Socio-oceanography seeks to integrate social science research on human behaviours, activism, public attitudes, effective communication and local ocean knowledge with findings from natural and physical sciences. Our case studies foreground the need for integration as a vital component in creating a holistic understanding of the policies, actions and challenges that shape human relationships with oceans. This interdisciplinary collaborative approach is therefore essential to ensure coherent and accessible actions and messaging to tackle problems from multiple angles across the broad range of disciplines. Furthermore, community engagement with science, incorporating the views and expertise of wider society, is key to addressing what is a ‘bigger-than-science’ set of problems. This paper explores diverse definitions of ‘community’, identifies the various rationales for engaging with different communities in the context of socio-oceanography, reflects upon how we currently engage with communities and importantly how we could engage differently, before outlining a ‘socio-oceanography in society’ approach. We begin from the position that members of communities are not ‘empty vessels’ in need of education or outside intervention. To use the ‘banking concept’ as developed in relation to education by Friere (1968) [9], plastic pollution researchers do not need to ‘deposit’ information into communities – indeed, especially in low resource communities, it is important that scientists do not conflate lack of formal education with lack of knowledge. Equally we argue that it is crucial that plastic pollution researchers do not replace *depositing* with *extracting* from members of communities. Working with communities does not necessarily avoid power dynamics. Liboiron [22] emphasises that pollution is embedded in colonialism and what they term ‘dominant’ (rather than Western) science practices. Thus ‘creating knowledge is an act based on values where some interests are reproduced and others are not’ [22]. The CLEAR lab project provides a radical critique of dominant science practices in terms of which data are collected and how, as well as access to land, acknowledging the role of indigenous communities and attributing scientific publications equitably [6].

In this paper we examine the dynamics of community engagement in the abatement of plastic pollution within the framework of socio-oceanography, to exemplify the benefits of this approach in the context of a global environmental problem. To shed light on this approach in practice we reflect upon a series of international case studies which present original empirical data from a range of projects representing diversity in types of communities, actions, geographical locations and cultures.

## In the context of socio-oceanography - What is community?

‘Community’ is a term which is interpreted widely across a broad array of diverse scholarly disciplines, expressing nuanced meanings with some commonalities. Many scholars emphasise the characteristics of ‘community’ with a defined structure, place, and space [23, 42]. The sense of community is represented by meaningful attachment and interdependence, creating shared beliefs, values, and experiences among individuals. Across disciplines, academics have defined the sense of community using various elements that work together to generate a comprehensive notion of ‘what a community is’. In the fields of sociology, anthropology, psychology, urban studies, and political sciences, the term reflects the attributes that are necessarily embedded in human societies [25, 36].

Assigning a universal definition to the term “community” is arguably challenging, considering the diverse contexts within which it is applicable (here it is worth reflecting on similar problems highlighted by Michael Warner in relation to studying ‘publics’ [41]. Human and biological communities are dynamic and complex systems, capable of self-organising to adapt to change and inherently heterogeneous. In both contexts, the individuals and the community itself benefit from the interactions among various components. In ‘community research’ or community engagement in socio-oceanography, there is no single definitive answer. What community means, or which communities are targeted for engagement, depends on disciplinary perspectives including core research questions and theoretical approaches. Geographical proximity is no longer necessary in terms of community connectedness as people can feel a ‘sense of community’ and ‘attachment’ or ‘belonging’ regardless of their physical location. Here we suggest that it is useful to consider issues of technology and scale, and we thus conceive of community as coming into being in four ways, each creating a type of community which operates on different scales: geographical, practical, virtual, and circumstantial.

Geographical communities are found within the same spatial location in settlements – villages, neighbourhoods, regions. Geographical communities can be impacted by plastic pollution in the form of plastics washing up on their local shorelines and the source of this pollution is frequently not as a consequence of use or mismanagement by local people, but rather due to waste mismanagement by communities at some considerable geographical distance from them (See Case Study 4).

Communities of practice come into being through people’s professions, livelihoods, or issue-based interests. These communities are wider in scope and draw together those who may never interact in person. These communities can occupy different points along the plastics lifecycle; from petrochemical companies that source and trade in the fossil-fuel feedstocks that make plastic, to policymakers that regulate plastic production, to end users such as local fishing communities. Although our focus here is generally on communities as end users of plastics, it is important to note that solving the plastics problem will necessarily involve ‘upstream’ communities of practice, i.e. those manufacturing polymers and products before they enter the commercial supply chain. The fishing community is an example of an (end user) community of practice engaged in the plastic problem, and one which can be further subdivided by type (e.g., creel fishers, trawl fishers, dredging fishers). These communities of practice are key to addressing plastic pollution bycatch during their activities, and from abandoned, lost, and otherwise discarded fishing gear (ALDFG) (See Case Studies 2 and 5)

Virtual communities, in contrast to geographical communities and communities of practice, initially come into being across digital platforms, rather than face-to-face interactions or livelihoods. Virtual communities are frequently built around, and (re)produced by social media platforms via groups or forums using hashtags, posts and reposts, or through popular messaging platforms. They may be specifically virtual and hosted entirely online or may overlap with other types of community. Communication technologies now enable communities of all kinds to operate as hybrids, spanning both geographical and virtual spaces and bringing ‘experts’ and ‘lay’ users together around a specific issue such as plastic pollution in novel ways. For example, the interactive online plastics ‘myth busting’ tool connects virtual communities of lay publics, scientists, activists, policy makers, journalists and industry (Case Study 1).

To these we can add a final layer: communities of circumstance. These are communities which may encompass characteristics of all the other types, and that form around external events or threats. Here, we could consider natural or anthropogenic disasters as creating communities of circumstance. With respect to plastic pollution, communities of circumstance may form in response to being impacted negatively by a specific event. For example, in May 2021 the largest marine plastic spill to date occurred when the M/V X-Press Pearl cargo ship caught fire 18 km off the west coast of Sri Lanka, spilling ∼1680 tons of pre-production plastic pellets or ‘nurdles’ into the surrounding water [7]. Huge volumes of nurdles are still now washing up daily several years after the spill, particularly affecting Sri Lankan coastal communities. This has been catastrophic for the fishing and tourism industries, and members of geographical communities have led on clean-up operations and monitoring efforts [16, 37, 43]. We would argue that interventions shaped and led by the priorities of community members are crucial to their success (See Case study 2).

It is important to note that these four defined community types are not necessarily distinct nor static but may come to share attributes at certain points. For example, contemporary communities of circumstance also frequently come into being virtually, where media platforms provide a useful focus for single-issue campaigns. These might concern conservation of marine resources e.g. lobbying against specific proposals for fish catch quotas, or towards protected area legislation. With respect to plastic pollution, communities can play an influential role in lobbying governments relating, for example, to tackling plastic waste, and bans on specific products such as single use items or intentionally added microbeads.

Communities of all types play central roles in resource management and environmental protection. Geographical communities and communities of practice have recognised rights and interests in the marine environment. They also have significant knowledge and skills relevant to socio-oceanography. They may have influence over what happens in the coastal zone, shaping coastal processes, marine pollution, fishing effort in the inshore and beyond. People may also be members of more than one relevant community simultaneously: consider indigenous scientists who are both scientists and indigenous knowledge holders (Wheeler and Root‐Bernstein 2020). Given how communities develop, evolve and frequently overlap, socio-oceanography approaches for engaging with communities must not therefore assume or impose ‘community’ and where possible the means of engagement should be co-designed from the outset. Throughout this paper we are using the definition of socio-oceanography as set out by the scientists who first developed the term [31] to draw upon the tools of natural and social sciences in exploring connections which may otherwise be marginalised or erased entirely due to disciplinary differences.

**Table:** 

***Case Study 1****: Mythbusting Plastics – An online tool to engaging with Communities using gamification – Lesley Henderson (Plastic Mythbusters)*
Funded by the UKRI Industrial Strategy Challenge Fund’s Smart Sustainable Plastic Packaging Challenge (SSPP), and in collaboration with the Hereon Institute of Coastal Environmental Chemistry and communications experts, we launched an online resource “Plastic Mythbusters” which challenges popular plastic myths in media. This involved an interdisciplinary academic team who bridged material and social sciences. Interviews conducted with households in different parts of the UK, plus Spain and Germany (n=34) revealed high levels of confusion about the sources of, and risks associated with, plastic pollution. Much of this was rooted in media reporting. These images of plastic pollution are emotive and powerful, reaching vast numbers of people and shaping public ideas about risks and solutions. For example, the way in which the media presents the issue of plastic pollution can shape the preference for certain solutions and sideline others. Many people believe that the Great Pacific Garbage Patch is a solid mass. Framing the problem in this way assumes that plastic pollution can be removed from the ocean with the correct technology – no need to change consumption behaviour or limit plastics production. However scientists describe the Great Pacific Garbage Patch as a “growing plastic smog” that does contain larger plastic items but is composed of micro and nano plastics over large distances not necessarily visible to the human eye. The online tool provides an interactive approach to dispelling these types of myths, which were rooted in the data emerging from the interviews.
In this way we know the tool is addressing genuine recurring myths which circulate within different communities. We are working closely with different leading microplastics scientists to replace myths with scientifically accurate information about plastic pollution which is compelling, but also openly acknowledges uncertainty. For example we are clear about the state of science where there is a lack of robust data – e.g. solving plastic pollution with “plastic eating bugs”.
Gamification is a positive way to engage different communities. Users represent a range of communities including lay public, policy makers, business and industry, NGOs and charities and academic communities (STEM and non-STEM) from diverse parts of the globe and include members of media. This initiative need not to be restricted to plastic pollution and works across a range of newsworthy and contentious scientific topics.

## In the field of socio-oceanography - Why should scientific researchers engage with communities?

In line with the definitions above, scientists form a large community of practice in themselves, with non-scientists or ‘stakeholders’ traditionally excluded from this ‘scientific community’. Scientists also form smaller communities, for example those working within defined fields, on specific projects, or towards a common research outcome. Socio-oceanography thus represents a newly emerging multidisciplinary academic community. For the purposes of this discussion, we are considering community engagement as that undertaken by academic researchers with those typically categorised as outside of the scientific community. Here it is important to note that this is a perspective, and as acknowledged earlier we are alert to the traditional privileging of formal scientific knowledge over lay understandings and tacit knowledge, an unproductive viewpoint which hinders effective action. We must also bear in mind that those being engaged in the context of specific ‘non-scientific’ communities will have highly relevant location-specific knowledge or expertise regarding changes in local conditions which is valuable to scientific communities. Crucially scientists should not automatically assume they can access this knowledge or expertise nor should they assume that the non scientific community is unable to engage with concepts of sampling or measurement. As Liboiron [22] points out, fishers are after all expert samplers who keep logs of their catch with dates, weather conditions and other environmental factors over considerable time frames. We therefore argue that the possible forms of engagement are varied, and we explore some common key motivations for engaging, with a specific focus on plastic pollution below.

### To learn

Community engagement provides the only means of hearing and understanding community perspectives and priorities, which can guide current or future research and action. Furthermore, community support is vital for facilitating research (for example, interventions concerning discarded fishing gear would be limited and unproductive without the direct input and support of local fishing communities; Case Study 2). We are not arguing that communities will always have the time, resources, or motivation to engage with researchers. Case Studies 1 and 4 provide examples of overcoming challenges in engagement to gain buy-in from communities. Indigenous communities have rich, intimate ‘location specific’ knowledge about their local environment, where and when pollution accumulates and how it changes in certain conditions; Case Study 3 details engagement with different types of community and how we should be engaging in innovative ways specifically regarding co-design of research. Community members and researchers can share respective knowledge of the impacts of plastic pollution informed by lived and professional experiences. Researchers should also engage with communities to learn, for example, how and why they use certain materials and in which contexts, their disposal methods for handling everyday waste, and the wider contextual factors that are important to them (Case Study 4). These might also include community perceptions and local myths which impact on effective actions to reduce plastic pollution. For example, this could range from access to local infrastructure to lack of trust in the government to successfully manage waste. Engaging local communities from the outset of the research process to set priorities helps ensure that the research is relevant, sensitive and well-informed and can mitigate predictable pitfalls and challenges.

**Table:** 

***Case Study 2****: Engaging the Fishing Community in Collecting Lost Plastic Fishing Gear: Kerala, India - Sreejith Sreekumar*
Over the last 10 years, fishers in the Vembanad estuary of Kerala, India observed a substantial increase in the presence of plastic and particularly single use plastics, compared to fish during their daily operations. However, there were apparently low levels of awareness concerning environmental implications of plastic pollution, coupled with limited recycling or reuse options, and thus fishers habitually discarded damaged or end-of-life plastic fishing gear into the aquatic environment.
Our novel project aimed to actively involve and motivate the fishing community, a community of practice, facilitated by local administrators and cooperative societies, to collect plastics during fishing activities. Awareness meetings and workshops were conducted to discuss specific issues surrounding the accumulation of marine litter in fishing operations. The project tackled the significant challenge of disposal of plastic fishing gear after retrieval. The research team sought support from NGOs and private agencies, establishing a strong linkage between the research institute, fishers, local administrators, cooperative societies and plastic collectors. The focus was on raising awareness among fishers about the extent and impact of plastic pollution in the marine environment. As a result, fishers began storing the collected plastics at landing centres or shore areas where NGOs then retrieved the material for recycling.
The project was not without challenges. Initially, there was considerable reluctance among fishers to engage with the study as sorting plastics during fishing necessitated extra labour without immediate incentives. However, certain groups within the community recognised the importance of the cause, the shared concern of plastic pollution and the ultimate benefits to their fishing practice, leading them to proactively participate. As this new process became established and began to function effectively, it served as an inspiration for other fisher groups. Subsequently this grew momentum with a significant portion of fishers voluntarily joining the program, demonstrating a community-wide engagement in addressing this common issue. The success of this initiative has yet to be validated, however we note significant benefits across the collaboration network including fishers, local administrators, plastic collectors and research team. We therefore see this as an exemplar of 'snowballing' as a tool to engage communities in social change.

**Table:** 

***Case Study 3:**** Community engagement in beach litter research on a Scottish Island – Thomas Stanton and Deirdre McKay. 50 Years of Litter on Skye (50YOLOS) project*
In 1972, Gerald Scott published the first study detailing the pathways of beach litter to complex coastlines. Scott’s work was based on litter surveys of two beaches on the Isle of Skye, UK. Five decades later, we revisited Skye. We wanted to know how the social and environmental contexts of beach litter had changed over time, thus community engagement is core to our methodology.
We contacted Skye Beach Cleans (SBC). SBC is a community of practice – a small Skye-based group who coordinate, support and organise community members to collect beach litter. SBC has a significant online presence, drawing together both local residents and visitors concerned about plastic pollution. SBC members helped us identify beaches to visit. They also introduced us to members of Skye’s geographical - place-based crofting - communities to interview. We also learned about other communities of practice, such as that of creel fishing, and communities of circumstance – e.g. community campaigns against the siting of aquaculture operations. Fishing and aquaculture inevitably lose and/or discard materials that contribute to Skye’s beach litter problem. Over six months of community consultation, we learned that all these forms of community shape the social context for beach litter. This social setting was key to our understanding of the environmental context of beach litter.
The volume and nature of the litter mean that international survey methodologies (the OSPAR/MCS protocol) do not work on Skye’s beaches. The Scottish Islands Federation Marine Litter Working Group realised this several years ago. But many community members have an intimate understanding of the region’s industries, social, and environmental processes. They shared vital local knowledge of litter sources and pathways. Community participation in our fieldwork enabled us to identify sources of beach litter that the OSPAR methodology would have categorised as unsourced (for example, items specific to aquaculture). Our data analysis corroborated their information, enabling us to challenge orthodox marine litter research and policy.
As community contributions have co-produced our findings we have invited community members to review our research outputs routinely for accuracy and sensitivity and included community members as co-authors on conference proceedings [39].

### To gather environmental data using citizen science approaches

A significant opportunity to be gained from science communication and public engagement is for researchers to gather rich data that would otherwise not be available or possible to collect, in the form of citizen science. In the case of plastics, there are wide ranging opportunities for communities to gather scientifically robust environmental data. Examples include data on plastic litter abundance on riverbanks and shorelines alongside trained NGO staff (for example through UK-based NGOs such as Thames21 (https://www.thames21.org.uk/thames-river-watch/), Marine Conservation Society [27], and Planet Patrol [38]. Other, more independent, activities include using apps to monitor plastic waste (https://www.litterati.org/) and measuring the amount of plastic pellets on beaches (https://www.nurdlehunt.org.uk/). These data (and many others) far exceed the gathering capacity of employed researchers, providing valuable insights into abundance, distribution and characteristics of plastic debris. Where data themselves are difficult to collect in the field, projects might typically provide the opportunity for citizens to collect samples using set protocols which are subsequently sent to professional laboratories for analysis, thus contributing to the scientific process. Further social data can be collected on levels of community engagement, for example through recording the number of people engaging with organised beach cleans.

**Table:** 

***Case Study 4****: Community Engagement in Tackling Plastic Pollution in Jatirejo Village, Pasuruan, Indonesia – Nieke Monika Kulsum and Lesley Henderson, PISCeS: Plastics in Society-A Systems Analysis Approach to Reduce Plastic Waste in Indonesian Societies project*
Indonesia faces significant challenges in reducing its plastic waste inputs to the ocean due to its extensive coastline, large population and lack of formal waste management practices. Jatirejo village, located in Pasuruan, is a typical low resource (geographical) coastal community grappling with negative impacts of plastic pollution. Working in partnership with local community influencers and delivery partners on the ground we used focus groups, semi-structured interviews and observational/ethnographic research to identify the importance of gendered perspectives. Women in Jatirejo village play crucial roles in the household and local economy, frequently running *warungs* (local eateries/shops). Their daily activities make them both contributors to, and victims of, plastic pollution. *Warung* owners use single-use plastics for packaging, underscoring the need for sustainable alternatives. Additionally, women manage household waste and are key stakeholders in waste reduction programs. Engaging with women provides valuable insights into consumption patterns, waste management practices, and socio-economic barriers which can help develop more effective solutions.
Efforts by NGOs and the government to mitigate plastic waste include behaviour change campaigns, training, and providing waste management facilities but observational research revealed that recycling bins were often repurposed for household use i.e. to store rice rather than plastic waste. Traditional practices such as open burning and powerful cultural beliefs that the ocean can absorb unlimited waste, exacerbate the problem. One local myth, “*Suleten”* promotes the idea that burning diapers can be harmful to the baby which results in members of communities disposing of these in bodies of water.
Mobile phones are the primary means of communication, and social media platforms are potentially powerful tools through which to raise awareness and mobilise communities. Specific platforms are therefore arguably well-placed to disseminate information about plastic pollution, promote clean-up events, and share success stories. Effective strategies could include content relevance (creating engaging content that resonates with local youth, using local languages and cultural references), interactive campaigns (encouraging participation through challenges, quizzes, and contests) and educational resources (providing easy-to-understand materials explaining the impact of plastic pollution and practical steps for mitigation). Our exploration of consumption practices revealed that plastic pollution messaging must also be ‘entertaining’. Younger people and women are high users of TikTok, Instagram, and WhatsApp which allows us to draw upon social media norms and existing networks of consumption.

### To raise public awareness and empower

Within Western/Global North society, media messaging about plastic pollution has led to a series of misconceptions and myths surrounding this field, which are crucial to dispel. Facts and figures may be compelling and powerful in terms of media values (novelty, interest, shock factor) yet may not be balanced due to the incremental nature of scientific research and lack of certainty on definite outcomes [1], Keller and Wyles 2021). For example, science is not yet able to provide definitive figures on human exposure, or the absolute risk to human health from microplastics, yet media coverage often omits uncertainty in favour of presenting ‘facts’ (Case Study 1). Scientists must deliver evidence-based information and make clear where there is uncertainty. This involves engaging fully with a range of communities to support scientific and media literacy. This will ultimately empower people to make informed decisions for themselves and their circumstances. Here we must also recognise the rise in conspiracy theories and perception of media as misinformation and ensure that messaging is also adapted to reach ‘difficult to access’ communities who have turned away from mainstream channels, for example due to eco anxiety, fake news or information overload. Community engagement can also be a means to provide opportunities and empowerment to marginalised communities or those currently ‘without a voice’ (Case Study 5).

### To help focus public and funder attention on critical issues

In the context of plastic pollution, research is driven by a broad group of funding bodies including governments, national funding agencies, corporations, private utilities and more, in addition to individual philanthropic donations. Funders are generally motivated to fund research into fields that are high profile, of known societal relevance, or highly relevant to them and their business. Raising awareness of critical and specific environmental issues, and the potential risks of not undertaking the research, is vital to attracting funding. This is evident for plastics research, where funding has broadly increased in line with trends in raised public awareness, scientific understanding of the problem, and newly implemented policies (for example plastic bag tax, plastic cutlery ban and microbead bans) [17]. Given much of scientific and wider academic output is behind paywalls (for example within paid-access journals) and frequently written in language that is inaccessible to the lay reader, it is important that this is translated and disseminated more widely. This need is more pressing where research is publicly funded, for example through government-funded research councils, and thus the public have a direct stake in the outcomes. Where research can have equal benefit for society, the environment and the funder, this is of crucial importance to communicate across the breadth of communities. With sufficient time, effort and financial support, research across the broad academic and community-scale can lead to breakthroughs that can be used across multiple scenarios.

## In the sphere of socio-oceanography- How *Do* We Engage with Communities and How *Should* we Engage?

Engaging with communities throughout the research process – from research priorities to design, analysis and dissemination – requires careful consideration of the case-specific circumstances in which our research is placed. The ways in which we as social and environmental scientists engage with communities are as diverse as the communities with which we engage. Just as there is no single ‘community’, nor is there a single way of engaging best. While we cannot be prescriptive in the case of plastic pollution, it is crucial that we bear in mind that each community comes to the topic with differing levels of prior knowledge, expertise, and vested interests in terms of strategic outcomes. However, sound ‘rules of engagement’ should be predicated on the understanding that engaging with any community requires an awareness of the very specifics of that community. The level of awareness and understanding will vary depending on who researchers engage with, the specific focus of their research, and the amount of time they have worked with the community in question. In all cases, establishing this understanding in the early stages of the research process necessitates two-way dialogues between researchers and the communities they work with.

Traditional engagement formats between scientists and wider community include science-led presentations to groups, workshops, science fairs, school events and citizen science projects. These may be extended to different forms of media engagement including blogs, podcasts and online gamification tools (Case Study 1). We might also include fora to interpret and validate data, inclusive data-collection campaigns, as well as creative and participatory arts (Case Study 5). Participatory initiatives led by community champions can help engage communities and sensitise participants in distinct ways which may ensure deeper investment in the project. Novel forms of communication such as music videos or puppet shows may help to dispel anxieties on the part of participants (for example see Bowyer et al. [5] regarding participatory approaches to air pollution sampling in Kenya).

Within the broader field of socio-oceanography we consider the role of community engagement across the spectrum of academics and communities as having an integral role to play in meeting the challenge of plastic pollution. This arguably presents a series of distinct dilemmas: despite the acceleration of papers published on the issues of plastic, microplastic, and nanoplastic pollution, there is no scientific consensus on either the scale of the problem nor the nature of plastics’ impacts on human health and the environment. Furthermore, there remains uncertainty over the efficacy of various ‘solutions’ to the plastic waste crisis. For example, there has been significant public and/or policy support for downstream, technical approaches to plastic pollution clean-up, such as the high-profile initiative led by nonprofit Ocean Clean Up [13]. However, implementing expensive, invasive and energy-intensive technologies to recover floating plastic pollution is scientifically controversial and risks displacing more effective upstream measures such as reduction of plastic production or interception of plastics at source, or at least before they reach the oceans [3, 4, 29]. This means that there is no simple message to convey to communities, and the message they receive must be shaped in a nuanced manner which takes account of existing perceptions, priorities and practices.

Media can certainly play a strong role in pushing certain science issues onto the public agenda, contributing to science’s public image, influencing its legitimation, public support, and funding [1]. Yet certain aspects of plastic pollution-related communication may raise specific challenges for example regarding communicating risk and ‘uncertainty’. A prime example is the communication of human health impacts arising from the impact of micro- and nanoplastics, where our understanding of exposure of humans to plastics remains incomplete. When we consider the health implications, it is only recently that data has emerged linking human health conditions with exposure to micro/nanoplastic particles and their accumulation within bodily tissues [19, 33], with insufficient data yet on long-term consequences for health. Nonetheless, the absence of evidence (of harm) does not equal the evidence of absence (of harm), a fact that has previously been confused in this scientist-public discourse on plastics [21, 35]. As illustrated in Case Study 1, when these research gaps are extrapolated, plastics related myths can emerge which can shape public and policy understandings of the topic, and frame the problem in specific ways e.g. the claim that ‘we eat a credit card’s weight in plastic every week due to microplastic pollution’ Gruber et al. [10], subsequently discredited by Pletz [30].

Concerns regarding scientific uncertainty, shifting power relationships, imbalances and social justice between various communities are of considerable interest to plastic pollution researchers and there are clear implications for socio-oceanography tools and approaches to engaging with communities. This approach extends debates beyond the specifics of socio-oceanography to consider various pressing societal issues which can impact on how academic evidence is consumed or engaged/disengaged with by various communities. Case Study 4 provides an example of some of the challenges of engaging communities, and different ways in which this engagement can be achieved. Here it is important to note that communities do not exist ‘out there’ ready to be engaged by researchers, rather they are constituted by technologies, policymakers, researchers and more. Communities do not engage with scientific messaging in a vacuum and the landscape within which people encounter images and stories concerning plastic pollution has arguably never been more cluttered. As academic researchers we are not communicating *to* people but *with* people. It is essential that interactions are much more than two-way dialogues but instead rooted firmly in a ‘science in society’ model which builds on and extends key moments in science-public relations (Irwin and [44]. Recent funding initiatives by the National Science Foundation (NSF, USA) include supporting ‘place-based environmental research’ to promote a “different way of doing science”. The U.S. government has instructed federal agencies to incorporate traditional ecological knowledge (TEK) into research and policymaking, thus integrating indigenous knowledge and western science within a new ‘science community’ to develop new ways of collecting and managing research data [26].

Further to this, environmental research requires future planning with regard to legacy; an integral part of this relies on ensuring community autonomy, for example over the use and disposal of materials. Non-scientific communities are also likely to be far more effective in articulating their specific challenges to a wider audience in an accessible manner (Case Study 5).

**Table:** 

***Case Study 5.**** Who Knows It Feels It: A Waste Pickers’ Perspective for a Just Transition - Cressida Bowyer*
Waste pickers play a critical role in cleaning the environment, and yet they are frequently excluded from decision making processes which affect their livelihoods directly.
Our project involved collaborating with 12 waste pickers from the Kenyan National Waste Pickers Welfare Association, and the Nairobi-based Social Justice Centre for Travelling Theatre to co-develop a piece of Legislative Theatre: *‘Who knows it feels it: A waste picker's perspective for a just transition’.* Legislative Theatre is a form of theatre designed specifically for social change that is performed for audiences consisting of the public, but also (unusually for traditional community theatre) policy makers, business owners, political leaders and others with a role to play in implementing systemic and institutional change. Working beyond the typical boundaries of issue awareness and community building, Legislative Theatre allows participants to address obstacles and oppression they face with the audience, who use the stage to explore how to create systemic change. Over the duration of a 5-day workshop, the theatre-making researched the lived experience of waste pickers (a community of practice). The resulting play showcased the significant amount of shared knowledge that the waste pickers possess, and highlighted the struggles they confront in the face of systemic and institutional discrimination and ignorance. This provided a unique insight into their lives and their role in advocating for recognition, integration and inclusion.
The play was performed in Nairobi, Kenya to different audiences with a different focus of engagement – specifically these included the general public, the waste picker community, the recycling industry, and policy makers gathered for the third round of negotiations of the United Nations Environment Programme Global Treaty to End Plastic Pollution. Powerful performances gave many policy makers and industry representatives a new and holistic understanding of waste pickers and their lived experience of plastic waste. Crucially this also facilitated better understandings of a just transition, and how this may look as part of the Global Treaty. Importantly, the process of not only participating in, but creating and leading their own event, allowed waste pickers to feel valued, recognised and empowered.

Embedding community engagement into long-term research can be particularly effective. This could entail co-designing research with community groups (see Case Study 3) or providing science training for community members. These initiatives offer communities choice over how they engage in research, including opportunities to shape research agendas and to generate new data that evidence place-based community knowledge [28]. Integrating community members throughout the research process can result in methodologies that recognise cultural heritage and respect indigenous communities’ sovereignty over their data. As Liboiron [22] points out scientific field sites are “homelands” and science partnerships are not an entitlement but must be earned continually [6].

Respectful, ethical co-working requires both goodwill and time. A key challenge is that research funding is often short term, with timelines for tangible outputs such as reports and peer-reviewed publications, being tight. Thus, to successfully deliver engagement via co-design and community science requires a longer-term commitment from funders. Embedding community engagement requires longevity and long-term impact which may only be realised over a period of years, especially where relationships need to be built, and evidence-based changes in perception and behaviour are mapped.

## Where next for community engagement with socio-oceanography?

Throughout this discussion we have highlighted the importance of scientific and wider academic engagement with diverse communities. However, before engaging outside of academia, it is essential that scientists from both environmental and social disciplines are communicating effectively with each other, aligned in their messaging and working towards common outcomes. Without this strong collaborative basis, any wider communication risks lacking coherence, leading to (or perpetuating) mistrust in science from the wider public, especially for such a high-profile yet arguably contentious issue as plastic pollution. Socio-oceanography therefore has an important role to play as an emerging discipline in its own right.

Engagement has multi-directional benefits and must be recognised as such; it is not solely carried out for the researchers or for the community. Community engagement not only enhances the value of research, but is often essential to its implementation, providing valuable background knowledge, access to resources and facilities, and opportunities to collect data as well as bringing rich insights to analysis of data and implementation of actions necessary. When implemented effectively, sharing of joint purpose can become a strong and powerful tool for change. In the context of plastic pollution, this is a visible and tractable problem, acknowledged widely across society. This therefore provides ample opportunities for community action, in the process also raising awareness of wider environmental issues.

It is important to recognise that communities are diverse and must necessarily be engaged in different ways depending on the type of community, its composition, the research questions, and the specific situation. Learning from experience and drawing upon our case study material here is vital to inform future research activities and avoid top-down research which is led and directed only by researchers. If we are truly to progress, this is an opportunity to rework traditional hierarchies and place community engagement at the heart of socio-oceanography.

The case studies and discussion presented in this paper focus primarily on end users of plastic however we recognise that engaging with communities along the full length of the plastics life cycle is key. Innovators, industry and policymakers will play a significant role in ending plastic pollution, and upstream measures must be highlighted as one of the key drivers of change. Further, communication and engagement beyond traditional narrow ideas of the “scientific community” has never been more important given the saturated media environment in which misinformation can be distributed and shared with rapid ease.

## Data Availability

Data related to case study 1 have been deposited in the UK Data Service ReShare https://dx.doi.org/10.5255/UKDA-SN-857057. Data availability to support other case study material is on request by contacting individual authors regarding their specific study.
